# Comprehensive profiling of lysine lactylation in *Candida albicans* and exploratory analysis of fluconazole tolerance associations

**DOI:** 10.1128/spectrum.00810-25

**Published:** 2025-09-30

**Authors:** Yuying Huang, Nana Song, Danrui Jing, Weida Liu, Dongmei Li, Xiaowei Zhou, Xiaofang Li

**Affiliations:** 1 Department of Medical Mycology, Hospital for Skin Diseases, Institute of Dermatology, Chinese Academy of Medical Science & Peking Union Medical College, Nanjing, Jiangsu, China; 2 Institute of Dermatology, Sichuan Academy of Medical Sciences and Sichuan Provincial People's Hospital 89669, Chengdu, China; 3 Institute of Dermatology, Chinese Academy of Medical Science, Jiangsu Provincial Key Laboratory of Dermatology, Nanjing, Jiangsu, China; 4 Department of Microbiology & Immunology, Georgetown University Medical Center 12231, Washington, DC, USA; 5 School of Life Sciences, Nanjing University 12581, Nanjing, Jiangsu, China; Rowan University Cooper Medical School, Camden, New Jersey, USA

**Keywords:** lysine lactylation, *Candida albicans*, tolerance, virulence, pathogenicity, post-translational modification (PTM), lactylome

## Abstract

*Candida albicans* is the primary pathogen of invasive candidiasis in most regions worldwide, but the therapy options for *C. albicans* infections are limited, and drug tolerance further exacerbates the treatment challenges. Lysine lactylation (Kla), a recently identified post-translational modification (PTM), is observed in numerous organisms; however, the role of Kla in *C. albicans* remains unknown. Hence, we report the first proteomic analysis of this specific modification in *C. albicans* and discuss its potential roles in drug tolerance of *C. albicans*. Altogether, 7,233 lactylation sites on 1,608 lactylated proteins were identified in *C. albicans*, with the highest degree of lactylation among the species studied so far. The further bioinformatics analysis revealed that the lactylated proteins were implicated in a variety of cellular functions with diverse subcellular localizations. Additionally, we found a unique survival mode of tolerant cells in the presence of fluconazole, which will be subject to a more thorough investigation in our future studies. This paper is the first report on the lactylome of *Candida* spp. and provides a reliable foundation for further research on Kla in *C. albicans* and other human pathogens.

**IMPORTANCE:**

This is the first report on the lactylome of *Candida* spp., and it provides some valuable insights for further research on lactylation in *C. albicans* and other human pathogens. Moreover, the observations in tolerant cells have prompted plausible hypotheses regarding the potential role of lactylation in mediating *C. albicans* tolerance to fluconazole, thereby offering a conceptual framework for subsequent investigations. Notably, fungal tolerance to azoles, a concept distinct from resistance, represents a critical phenomenon in *C. albicans* with profound clinical implications, as it directly correlates with therapeutic failure and persistent infections.

## INTRODUCTION

Invasive *Candida* infections are a serious threat to human health, with approximately 700,000 cases reported worldwide annually, which are seen as a common cause of bloodstream infections in intensive care unit patients. *Candida albicans* remains the primary pathogen of invasive candidiasis in most regions worldwide (1, 2). They are commensal organisms in the healthy human body, and when the integrity of the skin barrier is compromised and/or host immune function is weakened, they can migrate across epithelial tissues and enter the bloodstream, causing invasive infections and high fatality. Mortality rates of candidemia have been reported to be 40% to 70% in adults and 20% to 34% in neonates (3).

Currently, the treatment options for *C. albicans* infections are mainly limited to azoles, polyenes, and echinocandins, but with the spread of clinical use, more and more drug-resistant isolates have been reported. Moreover, there are often cases of delayed recovery and recurrence, even when using sensitive drugs in patients with persistent infections. This phenomenon is particularly prevalent among immunocompromised patients, in whom persistent candidemia may ultimately develop. The mortality rate of persistent candidemia is significantly higher than that non-persistent infections, which present greater challenges in controlling systemic infections (4). This discrepancy between clinical efficacy and low resistance levels might result from antifungal drug tolerance, which has been defined as the ability of drug-susceptible fungal strains to grow in the presence of antifungal drugs at concentrations above the minimum inhibitory concentration (MIC) (5, 6). In the literature, the terms resistance and tolerance are often employed interchangeably. Nevertheless, stable MIC elevation caused by irreversible genetic mutations is the main mechanism and manifestation of drug resistance, while drug tolerance is a manifestation of phenotypic heterogeneity intrinsic to a given isolate. Currently, the mechanisms underlying antifungal tolerance are recognized to be complex and dynamic, involving reversible epigenetic modifications, aneuploidy, and chromosomal duplications. Among these, reversible epigenetic regulation is still considered by several authoritative studies to play a central role in mediating tolerance, yet the specific regulatory processes remain unclear (6, 7).

In recent years, epigenetics has emerged as a prominent research focus in cancer, inflammation, and neurodegenerative diseases (8–10), yet it remains insufficiently studied in human fungal infection. Epigenetic modifications are reversible and dynamically regulate gene expression by specialized modifying enzymes (11). Compared with DNA epigenetics, protein PTMs influence gene expression through targeting amino acid residues like lysine in histone (12). The existing literature has documented over 300 types of PTMs, including methylation, acetylation, succinylation, phosphorylation, and crotonylation (13, 14). Recent studies demonstrated that PTMs are extensively involved in virulence regulation and metabolic adaptation in *C. albicans*, with enrichment observed in metabolic enzymes governing amino acid biosynthesis and carbon metabolic pathways. Specifically, key acetylated and 2-hydroxyisobutyrylated proteins have been demonstrated to participate in stress adaptation and ribosomal biogenesis (15–17). Our previous research further demonstrated that acetylation dynamically regulates the susceptibility of *C. albicans* to azoles, unveiling a transient upregulation of histone deacetylase genes *HDA1* and *RPD3* during early fluconazole exposure when MIC values remain sensitive (18).

Lysine lactylation (Kla), a novel PTM, is initially identified in mammalian core histones and directly promotes transcriptional activation analogous to acetylation (19, 20). Subsequent studies detected Kla in both histones and non-histone proteins across mammalian cells and phytopathogenic fungi (21). This PTM has been extensively characterized in cancer and neurodegenerative diseases, where it drives tumor microenvironment remodeling, immune evasion, and metabolic reprogramming to facilitate disease progression. Recent advancements in proteomic technologies have propelled the investigation into lactylation’s role in microbial pathogenesis. Emerging studies demonstrate that lactylation governs pathogenicity by modulating virulence determinants, immune evasion, and metabolic reprogramming in pathogenic microorganisms (21–23). However, the biological significance of lactylation in *C. albicans* remains entirely unexplored.

In this study, we systematically characterized the biological significance of lactylation in *C. albicans* and drug tolerance through enrichment of Kla-modified peptides coupled with high-resolution LC-MS/MS method. This work provides the first comprehensive view of the lactylome of *C. albicans*.

## MATERIALS AND METHODS

### Strains construction and drug susceptibility test

*C. albicans* strain Ca1, retrieved from an AIDS patient, was obtained from T. C. White (School of Biological Sciences, University of Missouri at Kansas City, Kansas City, MO, USA) and was grown on yeast extract-peptone-dextrose (YPD) agar medium. This patient experienced 14 episodes of oral candidiasis and was treated with progressively increasing doses of fluconazole. These isolates gradually developed into azole resistance over a two-year period and were documented in a series of 17 *C*. *albicans* isolates, with Ca1 being the initial isolate (24, 25). To collect tolerant cells of Ca1 (Ca1-T cells) and parental cells, we incubated strain Ca1 at 37°C for 48 h on YPD medium containing 32 µg/mL fluconazole (FLC) and on FLC-free control medium, respectively, before harvesting the samples (6, 26). MIC_50_ for strains was measured using CLSI M27-A4 guidelines. MIC_50_ was calculated at 24 h as the FLC concentration at which 50% of the growth was inhibited relative to growth in the absence of drug. SMG was calculated as the average growth per well above the MIC divided by the level of growth without drug at 48 h. RAD_20_ and FoG_20_ were measured using CLSI M44-A2 guidelines and *diskImageR* pipeline (the R script available at https://github.com/acgerstein/diskImageR/blob/master/inst/walkthrough.R) (6). E-tests were performed according to the manufacturer’s instructions with fluconazole E-test strips (Biomerieux). Ca1 was compared with *C. albicans* strain SC5314 in drug sensitivity tests, with each experiment replicated at least three times. Statistical analyses were performed using GraphPad Prism version 8.02, and SMG, RAD_20_, and FoG_20_ values were presented as mean ± standard deviation (mean ± SD).

### Protein extraction and digestion

The samples were collected and washed three times with 4°C phosphate-buffered saline (PBS) and then centrifuged at 6,000 rpm and 4°C for 10 min. The samples were ground to powder in liquid nitrogen and then transferred to 5 mL centrifuge tubes. Immediately afterward, 4 volumes of lysis buffer (8 M urea, 1% Triton X-100, 10 mM dithiothreitol, and 1% protease inhibitor cocktail, 3 mM trichostatin A [TSA; Sigma], 50 mM nicotinamide [NAM; Sigma], and 2 mM EDTA) were added to each tube of cell powder, and the samples were sonicated three times on ice with a high-intensity ultrasonic processor (Scientz). The remaining debris was centrifuged at 20,000 × *g* for 10 min at 4°C. Then, the protein was precipitated with cold (4°C) 20% trichloroacetic acid (TCA) for 2 h, followed by centrifugation at 4°C and 12,000 × *g* for 3 min, and the supernatant was discarded. The precipitate was washed three times by cold acetone. The protein was redissolved in 8M urea, and its concentration was determined using a bicinchoninic acid (BCA) assay kit (Beyotime). Protein solutions were reduced with 5 mM dithiothreitol for 30 min at 56°C and alkylated with 11 mM iodoacetamide for 15 min at room temperature in the dark. The samples were then diluted by adding 100 mM tetramethylammonium bromide (TEAB) until the urea concentration was below 2 M. Finally, the first digestion was performed overnight with added trypsin at a 1:50 mass ratio (trypsin-to-protein), followed by a second digestion for 4 h with trypsin added at a 1:100 mass ratio (trypsin-to-protein).

### TMT labeling

After digestion with trypsin, the peptides were desalted using a Strata X C_18_ solid-phase extraction (SPE) column (Phenomenex) and subsequently vacuum-dried. The peptide was then solubilized in 0.5M TEAB and handled following the manufacturer’s instructions for the TMT-10plex kit (90111, Thermo Fisher). The experimental design incorporated three biological replicates per experimental group to ensure statistical robustness. In the qualitative analysis, lactylation features were characterized using data from both parental and tolerant cells. In the quantitative analysis, lactylation differences were assessed by screening for proteins with differential lactylation modifications.

### HPLC fractionation

The tryptic peptides were first separated into 60 fractions using a gradient of 8% to 32% acetonitrile (pH 9.0) over 60 min and then combined into six fractions and dried by vacuum centrifugation. This process, called high-pH reverse-phase high-performance liquid chromatography (HPLC), was performed by an Agilent 300Extend C18 column (5 µm particles, 4.6 mm internal diameter [ID], and 250 mm length).

### Enrichment of lysine lactylated peptides

To enrich Kla-modified peptides, tryptic peptides in NETN buffer (pH 8.0, containing 100 mM NaCl, 1 mM EDTA, 50 mM Tris-HCl, and 0.5% NP-40) were incubated with prewashed antibody beads (lot number WM102; Micron Bio) overnight at 4°C shaken gently. Following incubation, the beads were washed four times with NETN buffer and twice with H_2_O. The bound peptides were eluted from the beads using 0.1% trifluoroacetic acid. Last but not least, the eluates were combined and vacuum-dried.

### LC-MS/MS analysis

The Kla peptides were dissolved and separated using a reversed phase analytical column (Acclaim PepMap RSLC C18 column, Thermo Scientific). The gradient comprised an increase from 2% to 10% solvent (0.1% formic acid in 98% acetonitrile) over 6 min, 10% to 20% over 45 min, and an increase to 80% in 7 min, followed by holding at 80% at least for 4 min, all at a constant flow rate of 250 nL/min on UPLC system. The peptides were subjected to nanospray ionization (NSI) followed by MS/MS in Q Exactive HF-X (Thermo Scientific) coupled online to a UPLC. The electrospray voltage was set at 2.0 kV. The intact peptides were detected in an Orbitrap instrument at resolutions of 70,000 and 17,500, respectively, with NCE setting of 30. Automatic gain control (AGC) was used to prevent overfilling of the ion trap. The m/z scan range was set from 350 to 1,600 for a full scan. The MS fixed first mass was set at 100 m/z. The affinity enrichment and LC-MS/MS analysis were conducted in Micrometer Biotech Company (Hangzhou, China). All changes in modification levels represent normalized results adjusted for variations in protein abundance.

### Database searching

MaxQuant with the Andromeda search engine (v.1.5.2.8) was used to analyze the raw MS/MS data. The tandem mass spectra collected were searched against the *C. albicans* strain_SC5314 database from the UniProt. Mass errors of fragment ions and precursor were set as 0.02 Da and 10 ppm, respectively. Trypsin/P was designated as a cleavage enzyme allowing up to four missing cleavage, five charges, and five modifications per peptide. Carbamidomethylation on cysteine was specified as fixed modification, and lactylation on lysine was specified as variable modification. The false discovery rate (FDR) thresholds for modification sites and peptides were set to 1%. The Kla site localization probability of >0.75 was excluded.

### Bioinformatics analyses

#### Annotation methods

Gene ontology (GO) of lactylation proteome was performed from the UniProt-GOA database (http://www.ebi.ac.uk/GOA/) based on three categories: cellular component, molecular function, and biological process. The functional domains of identified proteins were annotated by InterProScan (a sequence analysis application: http://www.ebi.ac.uk/interpro/) based on protein sequence alignment, with the InterPro domain database. The soft WoLF PSORT, an updated version of PSORT/PSORT II, was used to predict the subcellular localization of the lactylated protein in *C. albicans*. Protein secondary structures (β-strand, α-helix, and coil) were analyzed using the online tool NetSurfP, and the context sequences of amino acids around lactylated lysine residues (10 amino acids upstream and downstream of the site) in all protein sequences were analyzed by the software motif-x. The Kyoto Encyclopedia of Genes and Genomes (KEGG) database was used to annotate protein pathway, while the Eukaryotic Orthologous Groups (KOG) annotation of the proteome was derived from the NCBI-COG database (https://www.ncbi.nlm.nih.gov/COG/). The Cytoscape software was used to visualize the protein–protein interaction network, which was obtained from the STRING database.

#### Functional enrichment analysis

A two-tailed Fisher’s exact test was used to verify the enrichment of lysine lactylated proteins against all identified proteins. All projects with a corrected *P* value <0.05 are considered significant.

#### Enrichment-based clustering

For further hierarchical clustering based on differentially expressed protein functional classification, cluster membership was visualized by a heatmap using the “heatmap.2” function from the “gplots” R package.

### Western blots

A total of 20 µg of protein sample was mixed with 4× Sample Buffer, adjusted to 1–2 mg/mL, and denatured at 95°C for 10 min. Electrophoresis was performed using SDS–PAGE at 80 V for 30 min, followed by 120 V until the dye front exited the gel. Proteins were transferred to NC membranes at 200 mA for 1 h at 4°C. Membranes were blocked with 5% non-fat milk in TBST for 1 h at room temperature and then incubated overnight at 4°C with primary antibodies diluted in TBST containing 2.5% BSA. After washing, membranes were incubated with secondary antibodies for 1 h at room temperature. Signals were detected using HRP substrate and visualized by chemiluminescence, with exposure times adjusted according to signal intensity.

## RESULTS

### Susceptibility and tolerance of *C. albicans* strain Ca1

Through broth microdilution assays, the MIC_50_ of Ca1 parental cells was 0.125 µg/mL, whereas the MIC_50_ of Ca1-tolerant cells (Ca1-T) was identical to that of the parental cells. The supra-MIC growth (SMG) of Ca1, quantified at 48 h, was consistent with the observed trailing growth of Ca1. Through disk diffusion assays, RAD_20_ of Ca1 was consistent with MIC_50_, and FoG_20_ of Ca1 was consistent with SMG (Table 1; Fig. S1). E-test was used to validate the tolerance indicators measured by the above two methods, as shown in Fig. S2.

**TABLE 1:** The results of drug sensitivity tests^*a*^

Strains		MIC_50_ (µg/mL)	SMG	RAD_20_ (mm)	FoG_20_
Ca1	Ca1 parental cells	0.125	0.49 ± 0.007	17 ± 0.58	0.46 ± 0.01
	Ca1 tolerant cells	0.125			
SC5314		0.125	0.02 ± 0.004	17 ± 0.57	0.18 ± 0.01

^*a*^ MIC, Minimal inhibitory concentration; SMG, supra-MIC growth; RAD, radius of the zone of inhibition; FoG, fraction of growth; SMG, FoG_20_ and RAD_20_ values are expressed as mean ± SD.

### Proteome-wide analysis of Kla sites and proteins in *C. albicans*

In this study, 10,169 modification sites on 1,991 proteins were identified by LC-MS/MS analysis, of which 7,233 sites on 1,608 proteins had quantitative information, accounting for 10.99% of the total proteins (1,608/14,633) in *C. albicans* (27). Within the LC-MS/MS spectrum, we noticed a significant number of protein bands spanning a wide peptide mass range. Quantifying mass errors showed a near-zero distribution, mostly <10 ppm, indicating good data quality. Of these 1,608 proteins, 554 proteins contain only a single lactylation site, and 204 proteins contain more than 10 lactylation sites (Fig. S3). For example, the protein A0A1D8PF42, encoded by the gene *orf19.2296*, possesses the highest number of Kla sites, totaling 69. Additionally, there are 34, 12, and 3 lactylation sites, respectively, on heat shock protein 90 (Hsp90), Erg11, and Cdr1, which are proteins related to drug response.

To comprehend the overall distribution of lactylation in *C. albicans*, we contrasted the quantity of lactylated proteins identified in our study with those reported for other prokaryotes and eukaryotes, including fungi (*Botrytis cinerea* and *Phialophora verrucosa*), the rice (*Oryza sativa*) seeds, protozoan parasite (*Toxoplasma gondii* and *Trypanosoma brucei*), mouse Kupffer cells (KCs), and human hepatocellular carcinoma (HCC) cells. Based on the data, it is evident that *C. albicans* exhibits the highest number of lactylation sites and proteins (Table 2).

**TABLE 2:** Comparison of lactylated sites and proteins in *C. albicans* and other organisms

Species	No. of:		Reference
	Sites	Proteins	
*C. albicans*	7,219	1,608	This study
*P. verrucosa*	636	420	(28)
*B. cinerea*	273	166	(21)
*O. sativa* (rice seeds)	638	342	(29)
*T. gondii*	983	523	(30)
*T. brucei*	387	257	(31)
*Mouse* (Kupffer cells)	289	181	(32)
*Homo sapiens* (HCC cells)	2,045	960	(33)

Furthermore, subcellular localization prediction revealed a broad distribution of lactylated proteins, with cytoplasm proteins constituting the largest portion (40.86%), followed by proteins located in the cytoplasm and nucleus (23.69%), extracellular proteins (20.96%), and finally mitochondrial proteins (5.29%) (Fig. S3d). These findings underscore the diverse cellular distribution of lactylated proteins.

### Motif analysis of lactylated sites in *C. albicans*

**Fig 1:**
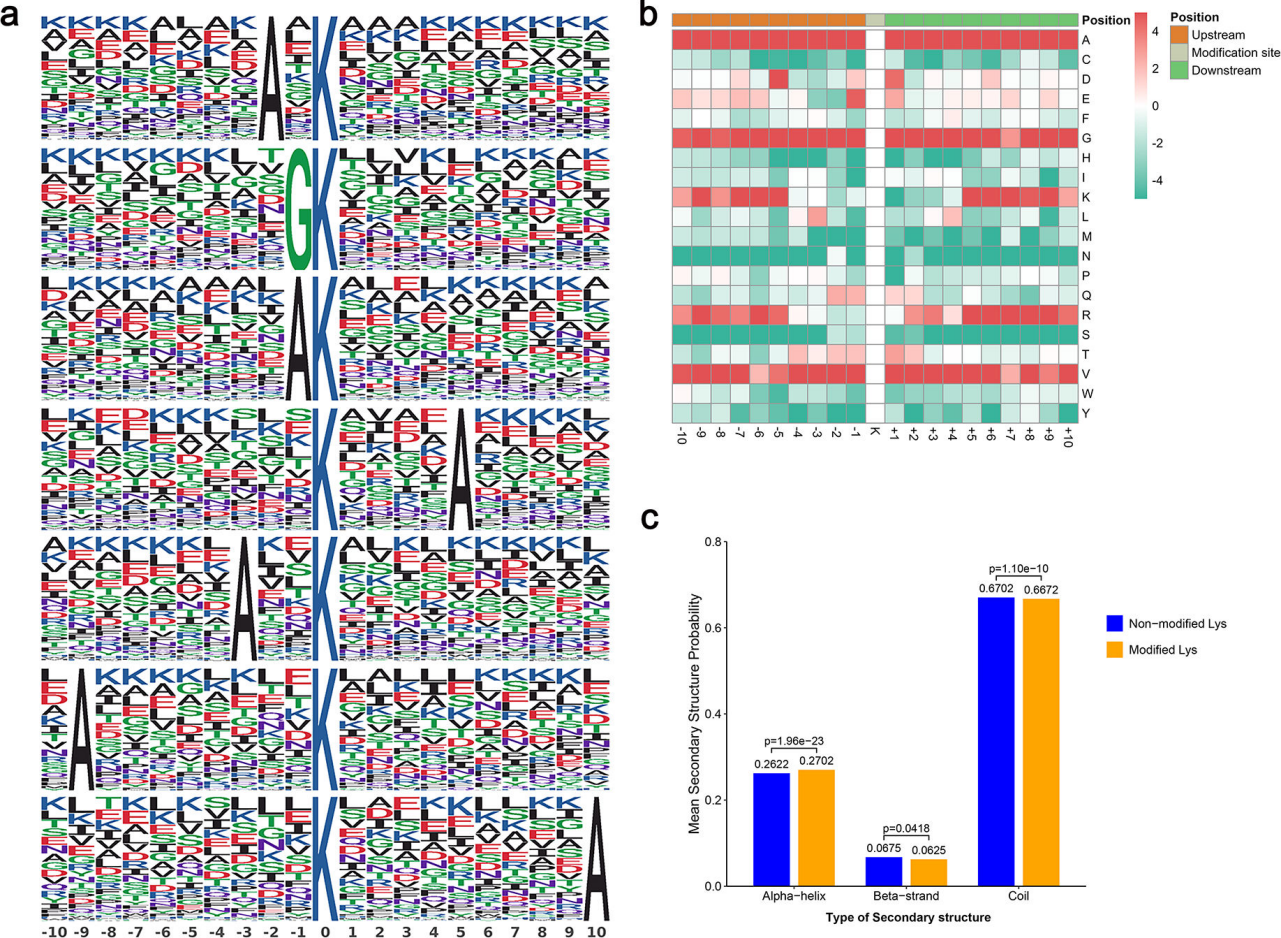
Sequence properties of the lactylated peptides. (**a**) Lactylation motifs identified by motif-x. The height of each letter corresponds to the frequency of that amino acid residue in that position. The central K refers to the lactylated lysine. (**b**) Heatmap showing the periodicity of the diverse types of amino acids nearby the lactylated lysine. The letters on the right side of the heatmap represent amino acids using single-letter codes. (**c**) Statistics of secondary structures of lactylated lysine and non-lactylated lysine.

To ascertain the sequence preference surrounding lactylated lysines, we performed motif analysis of amino acids located from −10 to +10 positions adjacent to lactylated lysine. The lactylated lysine contexts generated 25 conserved motifs as seen in Data S1. The top five motifs and their abundances are as follows: A^*^Kla (634 peptides), GKla (515 peptides), AKla (473 peptides), Kla^****^A (422 peptides), and A^**^Kla (375 peptides), where Kla signifies the lactylated lysine, and each asterisk represents a single amino acid residue (Fig. 1a; Data S1. The heatmaps of the amino acid sequences showed that lysine (K) was particularly liable to lactylation when the preceding amino acids are hydrophobic alanine (A) and neutral glycine (G). We also observed that A, G, and valine (V) residues were enriched in the −10 to +10 positions, while residues K and arginine (R) were significantly enriched in −10 to −5 and +5 to +10 positions (Fig. 1b). This signature parallels the sequence patterns observed in *C. albicans* succinylome and 2-hydroxyisobutyrylome (17, 34), suggesting that these PTMs may utilize the same enzymes for the initiation and reversal of these distinct modifications. In contrast, acidic amino acids, such as aspartic acid (D) and glutamic acid (E), predominate in lysine crotonylation of *C. albicans* (16), while K with positively charged amino acids predominate in Kla of other species (*B. cinerea*, *O. sativa*, and *T. gondii*). Additionally, in lactylated lysine proteins, approximately 27.02% of sites were positioned in α-helix, while 6.25% of sites contained β-strand structure, resembling the distributions observed in acetyl and crotonylation proteome of *C. albicans* (Fig. 1c) (15, 16).

### Functional enrichment analysis of lactylated proteins in *C. albicans*

**Fig 2:**
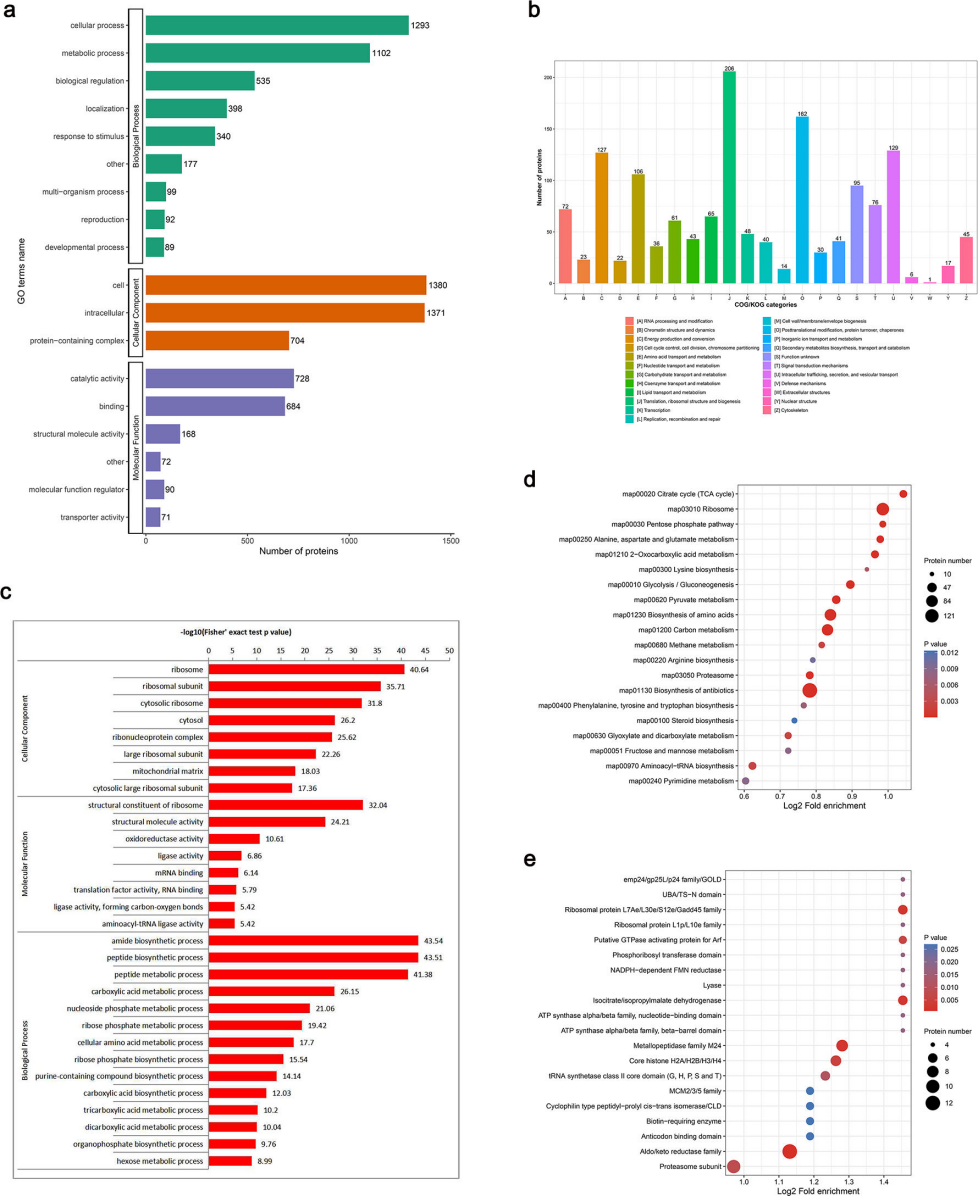
Functional enrichment analysis of lactylated proteins. (**a**) Function classification of lactylated proteins based on GO annotation. (**b**) Function classification of lactylated proteins based on COG annotation. (**c**) GO-based enrichment analysis of lactylated proteins according to cellular component, molecular function, and biological process. (**d**) KEGG pathway enrichment analysis. (**e**) The domain enrichment analysis.

To explore further biological regulations and functions of lactylated proteins in *C. albicans*, we conducted Gene Ontology (GO) and Clusters of Orthologous Groups (COG) annotations. With regard to biological processes, these lactylated proteins are mainly involved in the cellular process (80.41%), metabolic process (68.53%), and biological regulation (33.27%). As for molecular function, lactylated proteins are primarily enriched in two aspects: catalytic activity (45.27%) and binding (42.54%) (Fig. 2a). Classification based on COG showed that lactylated proteins broadly involved in translation, ribosomal structure (12.8%), PTM, protein turnover, and chaperones (10.1%) (Fig. 2b). Functional analysis of these modified proteins indicated that they typically modulate pathways that participate in translation, metabolism, biogenesis, and PTM in *C. albicans*.

Subsequently, we conducted functional enrichment analyses based on GO. The enrichment analysis of biological process revealed that the majority of lactylated proteins were implicated in peptide biosynthetic and metabolic process, amide biosynthetic process, and nucleoside phosphate metabolic process. According to the enrichment analysis of cellular component, lactylated proteins were primarily localized in the ribosome and mitochondrial matrix. The molecular function enrichment analysis provided evidence that a significant number of Kla proteins were associated with structural constituent of ribosome and oxidoreductase activity, supporting the aforementioned findings. In addition, we also found significant enrichments of ligase activity, translation-related binding, translation factor activity, aminoacyl-tRNA ligase activity, and other-related compound-forming ligase activity (e.g., forming carbon-oxygen bonds) involved in the process of protein translation, which play crucial roles in successfully accomplishing the protein synthesis (Fig. 2c). Functional enrichment revealed that Kla is substantial in ribosomal proteins, suggesting a potentially significant connection between lactylation and ribosomal functions.

In KEGG pathway enrichment analysis, lactylated proteins were enriched in ribosome, biosynthesis of antibiotics, biosynthesis of amino acids, and carbon metabolism. Additionally, lactylated proteins were also significantly enriched in essential metabolisms, including the TCA cycle, glycolysis/gluconeogenesis, and pyruvate metabolism (Fig. 2d). These findings indicate that lactylated proteins play an important role in the ribosome pathway and mitochondrial metabolisms. The domain enrichment analysis further revealed that aldo/keto reductase family, metallopeptidase family (M24), ribosomal protein (L7Ae/L30e/S12e/Gadd45 family), isocitrate/isopropylmalate dehydrogenase, and core histone (H2A/H2B/H3/H4) were highly enriched in the *C. albicans* (Fig. 2e). The results indicated that lactylated proteins are extensively involved in translation, metabolism, and energy production.

### The lactylated lysine in histones of *C. albicans*

**Fig 3:**
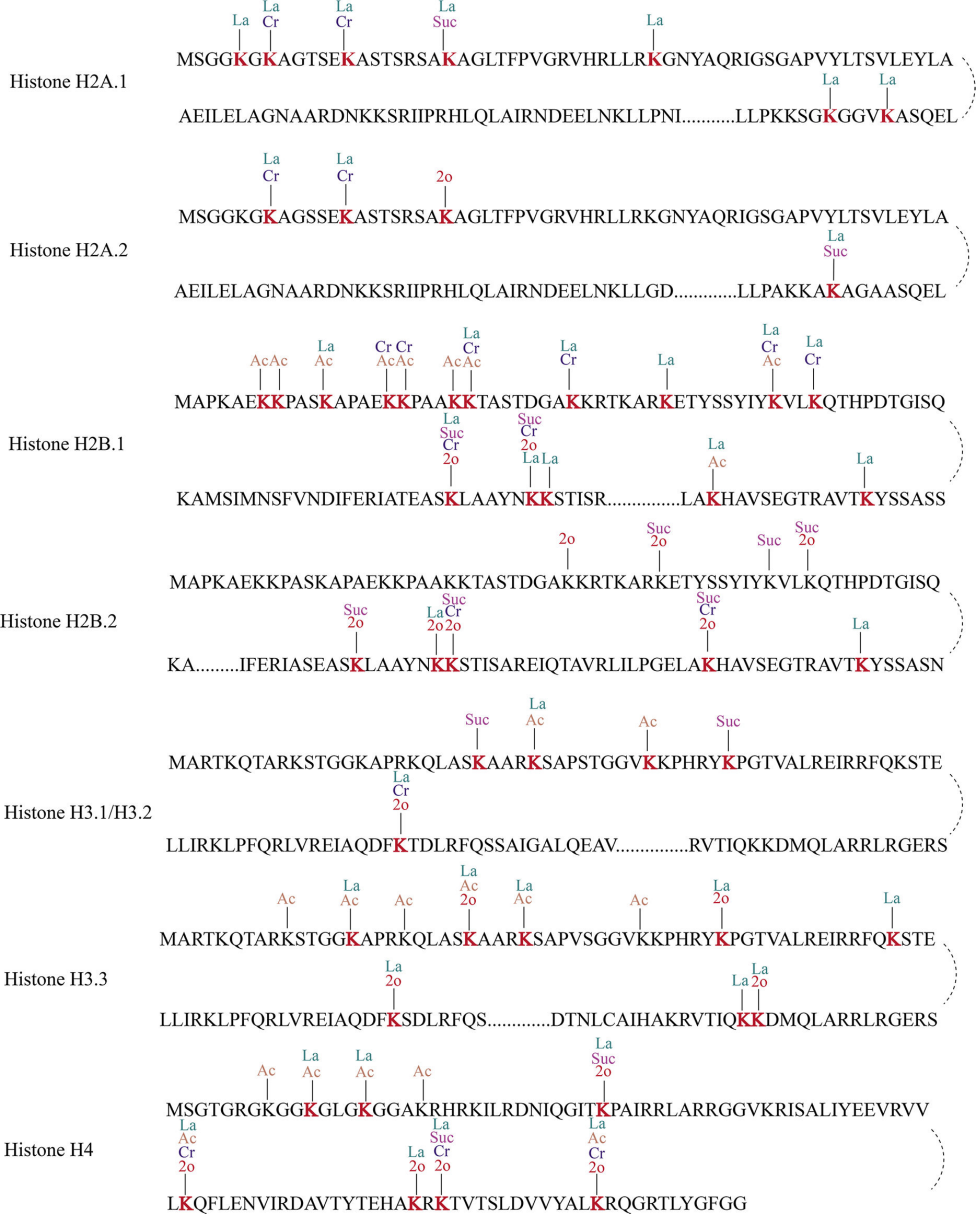
Post-translational modifications on histone proteins. La, Ac, Suc, Cr, and 2o represent lactylation, acetylation, succinylation, crotonylation, and 2-hydroxyisobutyrylation, respectively.

In previous studies, several eukaryotic and prokaryotic species have been identified to possess histone sites modified by lactylation, which were observed to directly enhance gene expression (19, 21, 29–33, 35). Our lactylome profiling revealed extensive lactylated histone proteins (particularly in H2A/H2B/H3/H4) within *C. albicans*. To explore whether Kla, lysine crotonylation (Kcr), lysine acetylation (Kac), lysine 2-hydroxyisobutyrylation (Khib), and lysine succinylation (Ksuc) can coexist on a single lysine residue and exert similar functions, we compared the lysine lactylome with the 2-hydroxyisobutyrylome, acetylome, crotonylome, and succinylome acquired in our previous research. To investigate potential functional crosstalk, we performed comparative analyses between lysine lactylation (Kla) and four major acylation modifications, including lysine crotonylation (Kcr), lysine acetylation (Kac), lysine 2-hydroxyisobutyrylation (Khib/K2o), and lysine succinylation (Ksuc), assessing their co-occurrence patterns at identical lysine residues. Interestingly, 30 out of 42 (71.4%) lactylated histone sites co-occurred with additional modifications, revealing that multiply modified lysine residues dominated over exclusively lactylated sites. As seen in Fig. 3, H2B.1 (K82 and K88) exhibits combinatorial Kla, Kcr, Ksuc, and Khib, while H4 (K61 and K93) carried Kla with Kac, Kcr, and Khib. Notably, evolutionarily conserved histone sites, including H2B.1K22/K46, H4K33, and H3K23/K79, exhibited multi-acylation patterns beyond lactylation. Particularly, H3K23—a well-characterized epigenetic hotspot—harbored concurrent Kla, Kac, and Khib in *C. albicans*, whereas H3K79, a conserved modification nexus, showed co-occurring Kla, Kcr, and Khib. This combinatorial modification network implies functional coordination of acylation crosstalk in regulating chromatin accessibility during specific biological processes in *C. albicans*.

### PPI network analysis of multiple PTM-modified proteins in *C. albicans*

**Fig 4:**
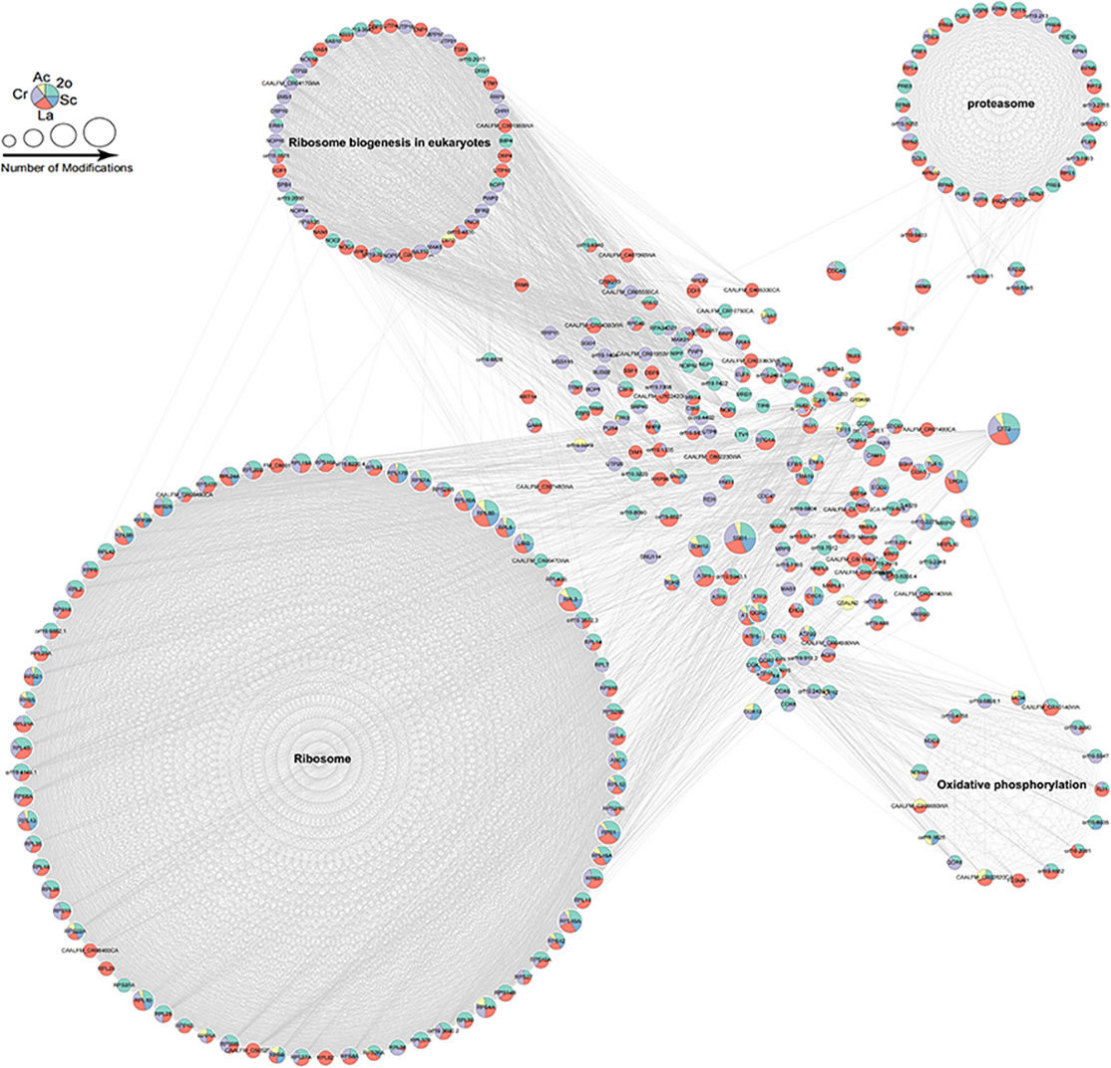
PPI network analysis of multiple PTM-modified proteins in *C. albicans*. Different colors of nodes represent differential modifications: lactylation (pink); acetylation (yellow); succinylation (blue); crotonylation (purple); 2-hydroxyisobutyrylation (green).

Based on acetylome, succinylome, crotonylome, and 2-hydroxyisobutyrylome, we conducted protein-protein interaction (PPI) analysis for lactylated proteins, resulting in the generation of 87 clusters (Fig. 4; Data S2). The PPI network used all lactylated proteins as nodes, directly linking them with 980 proteins through physical and functional interactions. Among these 980 lactylated proteins, 136 of them simultaneously contain four other PTMs. The most extensively modified 10 proteins are as follows: Hsp90, Ssa1, Eno1, Eft2, Cef3, Fas1, Ssb1, Ssc1, Met6, and Ssa2, which primarily perform translation activity and respond to stimulus and stresses.

We further demonstrated the occurrence of lactylation, acetylation, succinylation, crotonylation, and 2-hydroxyisobutyrylation in four greatly interconnected clusters and three key metabolic pathways: ribosome, oxidative phosphorylation, and proteasome. In these lactylated proteins, 74 ribosomal proteins are categorized into cluster 1, among which 22 ribosomal proteins contain five modifications, while 23 proteins participate in ribosome biogenesis and are grouped into cluster 2, with 16 proteins simultaneously undergoing 2-hydroxyisobutyrylation, crotonylation, and lactylation. Moreover, 27 lactylated proteins are grouped into cluster 3, associated with proteasome activity, and 16 lactylated proteins are categorized into cluster 6, closely related to oxidative phosphorylation. Interestingly, 85% (23/27) proteins are modified by more than three kinds of modifications simultaneously in cluster 3, while 88% (14/16) proteins are modified by three or more modifications in cluster 6. Many lactylated proteins are involved in multifarious cellular processes and PPIs. For example, ribosomal proteins Rpl82 and Rpl8b exhibit the strongest association with other proteins in cluster 1. Tdh3 in cluster 6 plays a crucial role in the oxidative phosphorylation pathway, while Rps3 in cluster 1 serves as a key component of the ribosome, playing a vital role in translation processes.

### Proteome-wide analysis of DLPs between Ca1 parental cells and tolerant cells

**Fig 5:**
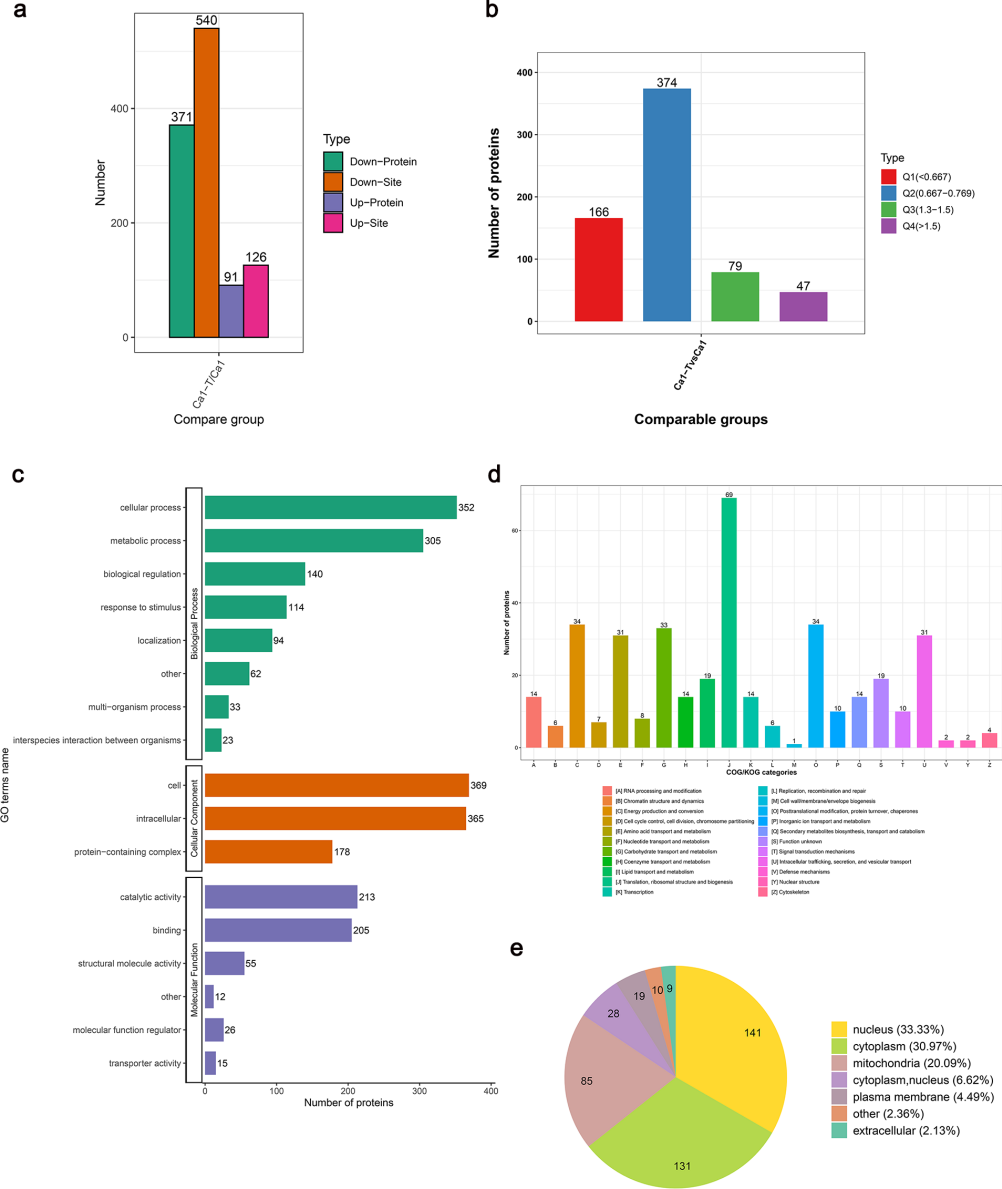
Basic analysis and functional classification of DLPs. (**a**) Statistical analysis of differentially lactylated proteins and sites. (**b**) DLPs are divided into four parts based on their fold change values, named as Q1 to Q4. (**c**) Function classification of DLPs based on GO annotation. (**d**) Function classification of DLPs based on COG annotation. (**e**) Subcellular localization prediction of DLPs.

We assessed the comprehensive protein lactate level and observed notable differences in lactylation between Ca1 and Ca1-T, indicating a potential role for protein lactylation in the development of antifungal drug tolerance. Altogether, 91 differentially lactylated proteins (DLPs) and 126 modification sites were upregulated, while 371 DLPs and 540 modification sites were downregulated in Ca1-T versus Ca1 (Fig. 5a). Then, we divided these DLPs into four groups based on their fold change values, named as Q1 to Q4. As shown in the figure, 47 proteins with significantly upregulated (>1.5-fold) and 166 proteins significantly downregulated (<0.667-fold) expression levels are noted (*P*<0.05) (Fig. 5b).

The biological functions affected by DLPs based on GO annotation revealed that the DLPs are primarily implicated in the cellular and metabolic process, biological regulation, response to stimulus, and catalytic activity (Fig. 5c). Classification based on COG revealed that DLPs were mainly concentrated in the translation, ribosomal structure and biogenesis, energy production and conversion, and post-translational modification, protein turnover, and chaperones (Fig. 5d). The subcellular localization prediction showed that DLPs mainly localize in the nucleus, cytoplasm, and mitochondria (Fig. 5e).

**Fig 6:**
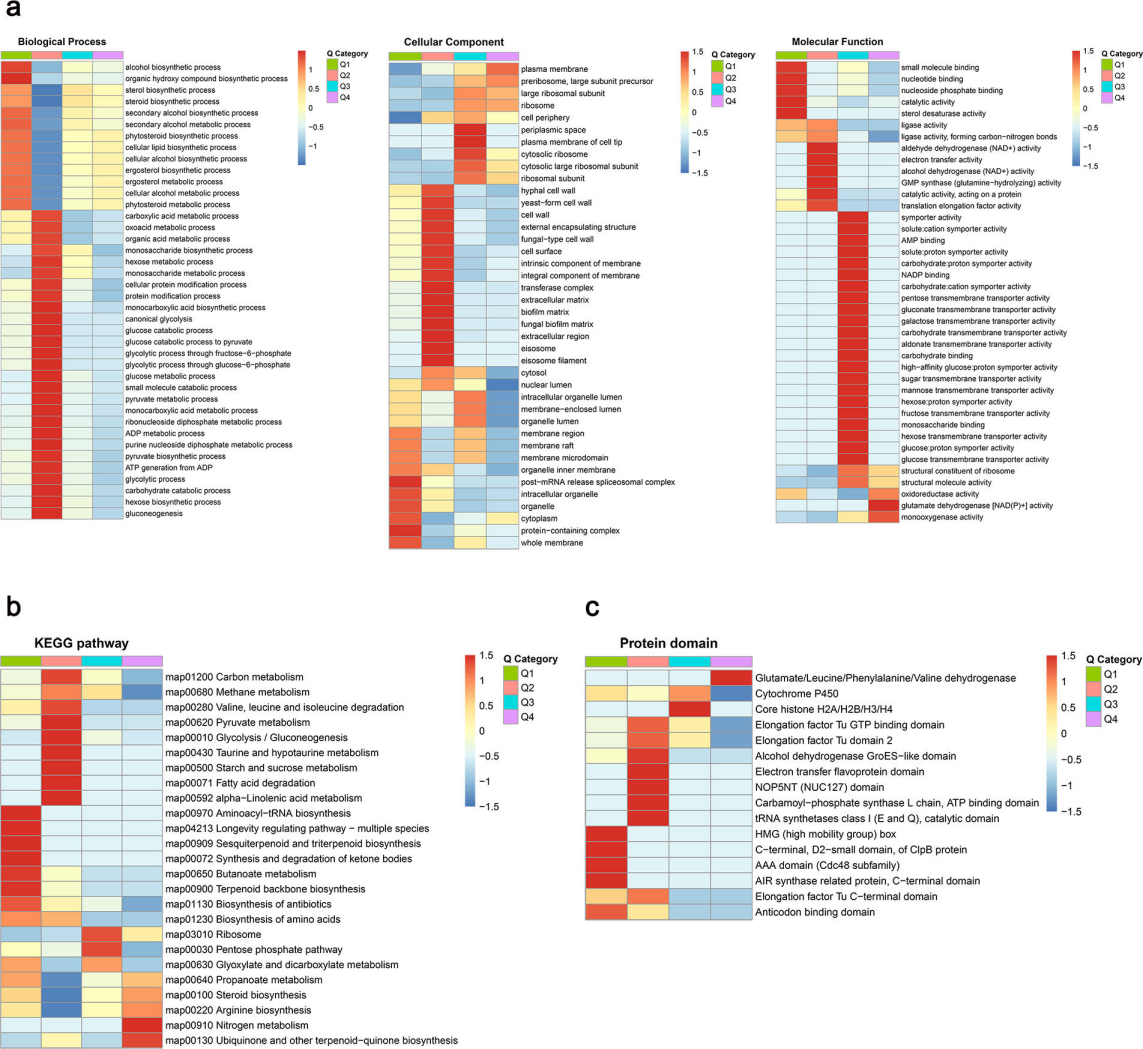
Functional enrichment-based clustering analysis of the DLPs. (**a**) GO enrichment-based clustering analysis of DLPs according to biological process, cellular component, and molecular function. (**b**) Clustering analysis based on KEGG pathway enrichment. (**c**) Clustering analysis based on the domain enrichment.

GO enrichment-based clustering analysis of the DLPs revealed that the significantly downregulated DLPs were predominantly related to alcohol biosynthetic and metabolic process, organic hydroxy compound biosynthetic process, sterol/steroid biosynthesis process, binding and catalytic activity, and post-mRNA release spliceosomal complex (Q1, <0.667-fold). Conversely, markedly upregulated DLPs showed enrichment in glutamate dehydrogenase [NAD(P)+] activity, monooxygenase activity, ribosome, and plasma membrane (Q4, >1.5-fold). DLPs in the Q2 group (0.667~0.769 fold) exhibited significant enrichment in organic acid metabolism, glycolysis/gluconeogenesis, pyruvate metabolism, and ATP generation, along with molecular functions, including electron transfer activity, aldehyde/alcohol dehydrogenase (NAD+) activity, and glutamine-hydrolyzing activity. DLPs in the Q3 group (1.3~1.5 fold) were primarily enriched in carbohydrate transmembrane transporter activity (Fig. 6a). The results of clustering analysis based on KEGG pathway enrichment are shown in Fig. 6b. The downregulated DLPs were mainly associated with glycolysis/gluconeogenesis, aminoacyl-tRNA biosynthesis, and carbon metabolism, while the nitrogen metabolism pathway, ubiquinone biosynthesis, pentose phosphate pathway, and ribosome were significantly upregulated in Ca1-T. These findings indicate that Kla might potentially regulate the homeostasis of energy metabolism by downregulating the glycolysis/gluconeogenesis and central carbon metabolism pathways and simultaneously upregulating the antioxidant stress response pathways, thus enhancing the cellular ability to survive under drug stress.

Clustering analysis based on protein domain enrichment demonstrates that DLPs associated with glutamate, leucine, phenylalanine, and valine dehydrogenase and core histone domains (H2A/H2B/H3/H4) are significantly upregulated, while the HMG (high mobility group) box, EF-Tu C-terminal, and AAA domain (Cdc48 subfamily) are markedly downregulated (Fig. 6c).

**Fig 7:**
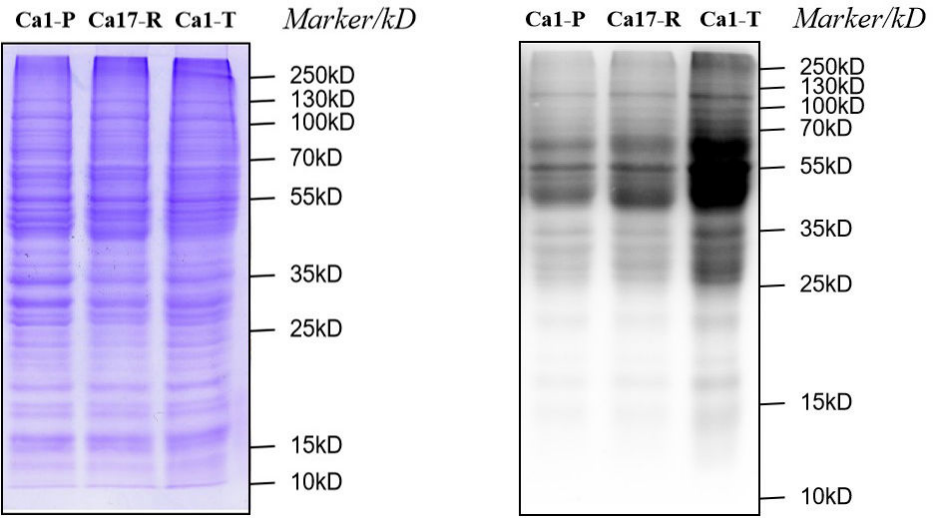
Coomassie total protein stains are shown at the bottom as loading controls. Western blot (WB) analysis of pan-Kla levels in three *C. albicans* strains: Ca1-P (parental cells, MIC = 0.125 µg/mL), Ca17-R (resistant cells, MIC = 64 µg/mL), and Ca1-T (tolerant cells, MIC = 0.125 µg/mL). WB results demonstrate a marked upregulation of global lactylation in the tolerant cells (Ca1-T) compared to both parental (Ca1-P) and resistant (Ca17-R) cells.

To further validate these findings, we performed immunoblot analysis using a pan-Kla antibody to compare lactylation levels among tolerant, parental, and resistant cells. The results revealed a significantly elevated lactylation level in tolerant cells relative to parental cells, while no significant difference was observed between drug-resistant and parental cells (Fig. 7).

## DISCUSSION

As a novel post-translational modification identified recently, lysine lactylation (Kla) has been proven to play vital roles in cancer, inflammation, and regeneration (36). However, there is limited research exploring its implications in the pathogenicity and drug sensitivity of human pathogenic fungi. This is the first study of lactylome in human pathogenic fungi. We analyzed the lactylated sites of *C. albicans* proteome and got the first systematic study of the lactylome from *C. albicans*. While the resistance mechanisms of *C. albicans* to fluconazole have been well characterized, investigations into its tolerance mechanisms toward azole antifungals remain in the preliminary stages. Particularly in immunocompromised patients, the emergence of azole tolerance significantly complicates therapeutic interventions and is associated with worse clinical prognoses. Notably, through comparative analysis of lactylation between tolerant cells (Ca1-T) and parental cells (Ca1), we have gained initial insights and formed some speculations regarding the role of lactylation in the mechanism of fluconazole tolerance in *C. albicans*. Compared to other species, *C. albicans* exhibits the highest number of lactylation sites and proteins (21, 28–33). The discovery of previously uncharacterized Kla proteins from various cellular compartments, possessing diverse cellular functions, suggests that lactylation plays a regulatory role in cellular pathways extending beyond DNA-templated processes.

Lactylated proteins widely exist in various cellular components and influence various biological processes within cells. They engage in diverse cellular functions, such as ribosomal biogenesis, translation, carbon metabolism, and PTMs. In our data set, we identified a total of 13 lactylated proteins and 106 lactylated sites on Elongation factor Tu (EF-Tu) protein family, which are GTPases that deliver aa-tRNAs to the ribosome in its GTP-bound form (37). Additionally, significant lactylation modifications were observed on proteins within the L7/L12 stalk and EF-G protein family (Fig. S4), which function as indispensable molecular switches in the process of protein translation (37–39). Based on these data, we speculate that lactylation modification may dynamically regulate the precision of ribosome complex assembly and translational fidelity, thus affecting the quality of protein biosynthesis in *C. albicans*. Moreover, lactylation was observed on multiple factors responsible for mRNA splicing, processing, export, translation, and degradation. Gene regulation and expression are predominantly regulated by transcription-related proteins, including 15 ribosome-binding proteins (RBPs), 4 DNA-binding proteins (DBPs), 5 RNA polymerases, and 223 RNA-binding proteins. These proteins are essential for pathogenicity and growth of *C. albicans* (40–42). Notably, Slr1—an SR-like RNA-binding protein implicated in virulence—was also found to be lactylated (40). The absence of *slr1* decreases *C. albicans* growth rate and leads to a defect in host cell damage *in vitro* and low virulence in a murine model of disseminated candidiasis (43). These observations raise the possibility that lactylation may contribute to the regulation of gene expression programs relevant to fungal adaptation and virulence.

Histone PTMs are key epigenetic regulators that influence chromatin structure and transcriptional activity, thereby modulating gene expression, genome stability, and antigenic variation (44, 45). In our study, we identified 42 Kla sites on *C. albicans* histones, a number considerably higher than those reported in humans (26 histone Kla sites) (19), mice (16 histone Kla sites) (32), *T. brucei* (16 histone Kla sites) (31), and *T. gondii* (20 histone Kla sites) (30). Notably, several histone lysine residues exhibited multiple co-existing PTMs, suggesting potential crosstalk between lactylation and other modifications such as acetylation and methylation. Among these, H3K79—a site previously implicated in transcriptional regulation^46^—displayed increased lactylation in tolerant cells, raising the possibility that Kla at this position may contribute to stress-adaptive gene expression. In parallel, lactylation of H3K23, which is recognized as a key epigenetic marker in mammalian systems, was also found to be significantly upregulated in tolerant cells (Fig. S5), supporting its potential involvement in antifungal adaptation. Furthermore, lactylation of H3K56 and H4K10 was also identified, with lactylation of H3K56 known to be regulated by glucose transporter 3 (Glut3) and playing a significant role in the invasion and metastasis of gastric cancer (47). Notably, lactylation of H4K10, previously unreported in other species, was downregulated in tolerant cells, suggesting that specific Kla marks may differentially modulate chromatin-related responses under antifungal pressure. These findings collectively point to a potential regulatory role of histone lactylation in the epigenetic control of *C. albicans* tolerance and pathogenicity, although further mechanistic studies are needed to verify these functional implications.

Apart from histones, numerous non-histone proteins associated with *C. albicans* virulence and drug responses were found to be lactylated. For example, 34 Kla sites were detected on Hsp90, a key factor of stress responses and tolerance. In tolerant cells, three sites on Hsp90 were downregulated, while one site was upregulated, suggesting site-specific regulation. Similarly, 69 Kla sites were identified on the protein A0A1D8PF42 (encoded by the gene *orf19.2296*), a mucin-like protein potentially involved in drug stress adaptation (48–50), with two sites upregulated and six downregulated in tolerant cells. Ssa1, a novel essential for epithelial cell endocytosis and systemic infection (51), harbored 40 Kla sites, most of which were downregulated in tolerant cells, indicating a possible role in modulating host–pathogen interactions. Mkc1, a MAPK pathway component linked to regulating hyphal and biofilm formation in *C. albicans* (52), was also lactylated. The protein A0A1D8PF90 (encoded by *orf19.5281*), a homolog of Scp160 involved in telomeric silencing and genome stability (53), exhibited 36 Kla sites. Disruption of the *scp160* is not lethal but results in cells of decreased viability, abnormal morphology, and increased cell ploidy and DNA content (53). Compared to parental cells, the lactylation at six sites on this protein was downregulated in tolerant cells. Furthermore, lactylation sites were identified on Erg11, Erg3, Erg5, Cdr1, and Mrr1, the proteins closely linked to drug resistance in *C. albicans* (54). Notably, Erg3 and Cdr1 showed decreased Kla levels in tolerant cells, while mixed regulation was observed on Erg11 and Erg5. These findings suggest that lactylation may influence multiple tolerance-related pathways. Finally, PPI network analysis indicates that lactylated proteins are likely to form cooperative modules, particularly in processes related to drug resistance and virulence of *C. albicans*.

**Fig 8:**
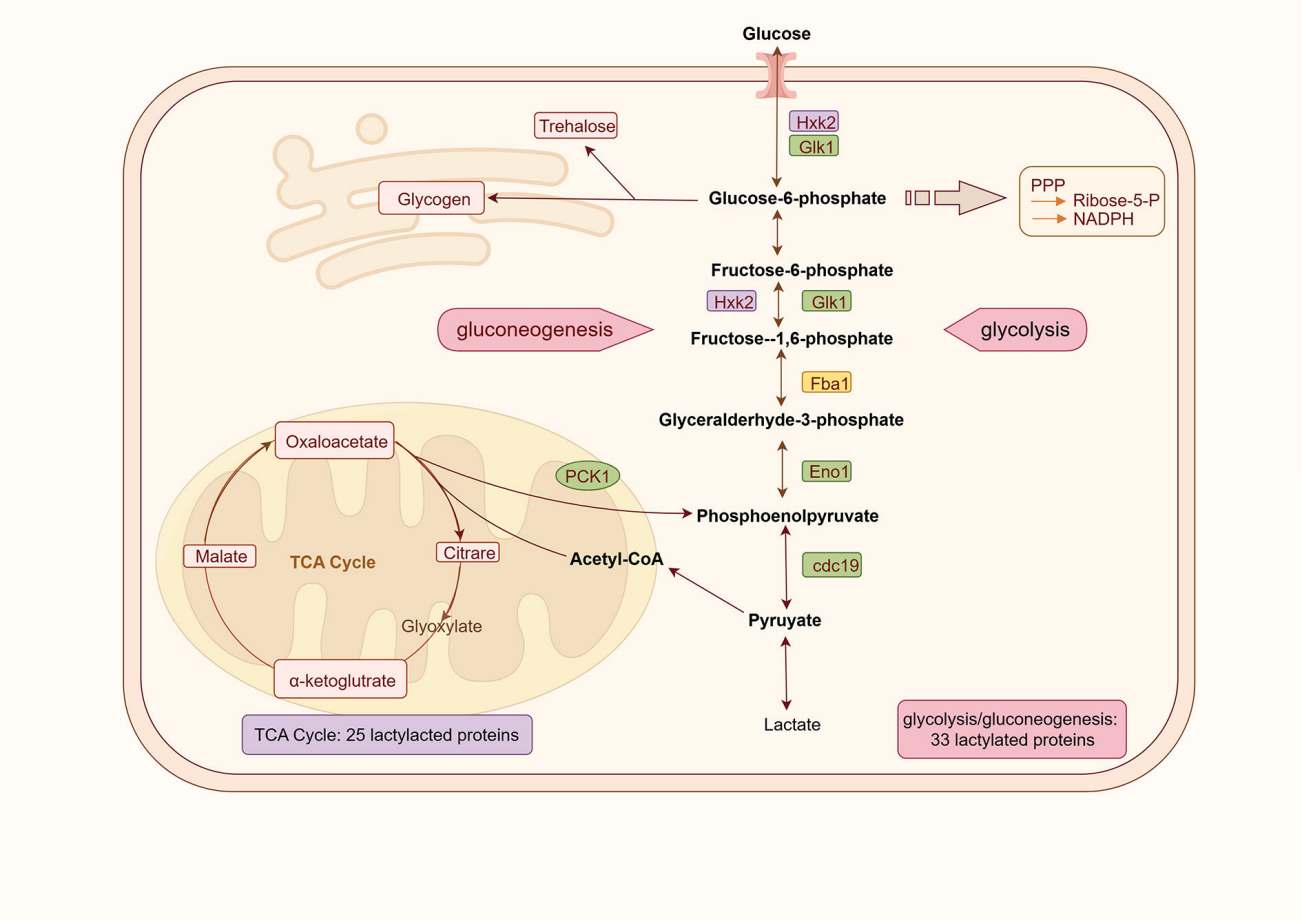
Lactylated enzymes involved in the glycolysis/gluconeogenesis and TCA cycle and associated with the virulence or drug resistance of *C. albicans*. Enzymes highlighted in purple indicate those that undergo lactylation but show no difference in lactylation between tolerant cells and parental cells. Enzymes highlighted in green are modified by lactylation and exhibit downregulated lactylation in tolerant cells. The enzyme highlighted in yellow indicates the presence of both upregulated and downregulated lactylation sites in tolerant cells.

Previous research demonstrates that enhanced glycolysis and lactate accumulation in cancer cells are proportional to the degree of lactylation, which is closely associated with tumorigenesis and drug resistance (19, 55). In our study, lactylated proteins were significantly enriched in central carbon metabolism pathways, including glycolysis, gluconeogenesis, and TCA cycle—key metabolic hubs for *C. albicans* virulence and viability. These findings raise the possibility that lactylation participates in transcriptional and metabolic regulation, consistent with findings in *Trypanosoma brucei* and rice (29, 31). The first step of the glycolytic pathway is catalyzed by hexokinase 2 (Hxk2) and glucokinase 1 (Glk1). Lactylation was detected on Hxk2 and Glk1 (Fig. 8), both of which are known to influence adhesion, filamentation, virulence, and pathogenicity of *C. albicans* (56, 57). Notably, Glk1 lactylation was downregulated in tolerant cells, suggesting a potential role in modulating drug tolerance via glycolytic flux and its own expression. Fructose-1,6-bisphosphate aldolase 1 (Fba1), another essential glycolytic enzyme implicated in invasiveness through its interaction with plasminogen (58), exhibited 15 Kla sites, with four downregulated and one upregulated in tolerant cells (Fig. 8). Other key glycolytic enzymes, including enolase 1 (Eno1) and pyruvate kinase (Cdc19), showed decreased lactylation levels in tolerant cells (Fig. 8). Given that mutants of these enzymes exhibit drug sensitivity and reduced virulence (59, 60), the downregulation of Kla may reflect an adaptive metabolic remodeling under fluconazole stress. Similarly, phosphoenolpyruvate carboxykinase 1 (Pck1)—a key enzyme of gluconeogenesis—displayed 11 Kla sites, with both the lactylation levels and protein expression showing a downward trend in tolerant cells (Fig. S6). Previous studies have indicated that *pck1* mutant strains exhibited reduced virulence but remained capable of infecting hosts (57). Within the TCA cycle, 276 Kla sites were identified across 25 proteins, with nine proteins showing decreased lactylation in tolerant cells. Lactylation of metabolic enzymes enables cells to respond to environmental changes by rapidly sensing their energy state and flexibly altering rates or directions (22, 61). The reduced central carbon metabolism and central energy metabolism were identified as the most characteristic features of the resistance (62). This widespread decrease in Kla across central carbon metabolism may serve as a feedback mechanism to limit energy metabolism under antifungal stress. Downregulation of energy metabolism and glycolytic capacity appears to be a hallmark of fluconazole tolerance, allowing *C. albicans* to survive slowly in a low-energy, drug-exposed state.

Further study will elucidate the mechanistic contributions of lactylation to the pathogenic adaptation of *C. albicans*, thereby advancing our comprehension of its role in drug resistance or tolerance, identifying lactylation-dependent pathways as potential therapeutic targets for future antifungal strategies.

### Conclusion

This is the first study to systematically delineate the lactyl-proteome of the human pathogen *C. albicans*. Our findings expand the existing understanding of the role of lactylation in protein modification and elucidate potential functions of this novel PTM in *C. albicans*. Considerable Kla modification, encompassing a variety of metabolic processes and biological regulations, underscores the importance of lactylation in modulating *C. albicans* physiology. Furthermore, differential analysis results demonstrated that DLPs are closely associated with biosynthetic and metabolic processes of *C. albicans*. Although our study provides an interesting perspective on the relationship between lactylation and drug tolerance in *C. albicans,* additional validation is necessary to fully comprehend the role and influence of lactylation in tolerance. Therefore, further investigations will delineate lactylation-mediated regulatory mechanisms underlying phenotypic tolerance and pathogenicity in *C. albicans*, aiming to provide innovative and potent therapeutic targets for refractory *Candida* infections in immunocompromised patients and offer insights into the lactylome studies of other pathogens.

## Data Availability

The mass spectrometry proteomics data have been deposited to the ProteomeXchange Consortium via the PRIDE partner repository (http://www.ebi.ac.uk/pride) with the data set identifier PXD056982.

## SUPPLEMENTAL MATERIAL

Data S1 spectrum.00810-25-s0001.xlsx Motif analysis of lactylated sites - 25 conserved motifs.

Data S2 spectrum.00810-25-s0002.xlsx Protein-protein interaction network-node.

Supplemental figures spectrum.00810-25-s0003.docx Figures S1 to S6.

ASM does not own the copyrights to Supplemental Material that may be linked to, or accessed through, an article. The authors have granted ASM a non-exclusive, world-wide license to publish the Supplemental Material files. Please contact the corresponding author directly for reuse.
