# Supplementary material for: Comprehensive profiling of lysine lactylation in Candida albicans and exploratory analysis of fluconazole tolerance associations
Source: Microbiol Spectr. 2025 Sep 30;13(11):e00810-25. doi: 10.1128/spectrum.00810-25 (PMC12584692; doi:10.1128/spectrum.00810-25)
Supplement: Supplemental figures — Figures S1 to S6. [file spectrum.00810-25-s0003.docx]

**Supplementary Materials**

**Supplementary Data 1** (The excel named ‘Supplementary Data 1’)

**Supplementary Data 2** (The excel named ‘Supplementary Data 2’)

**Supplemenary Fig. 1**


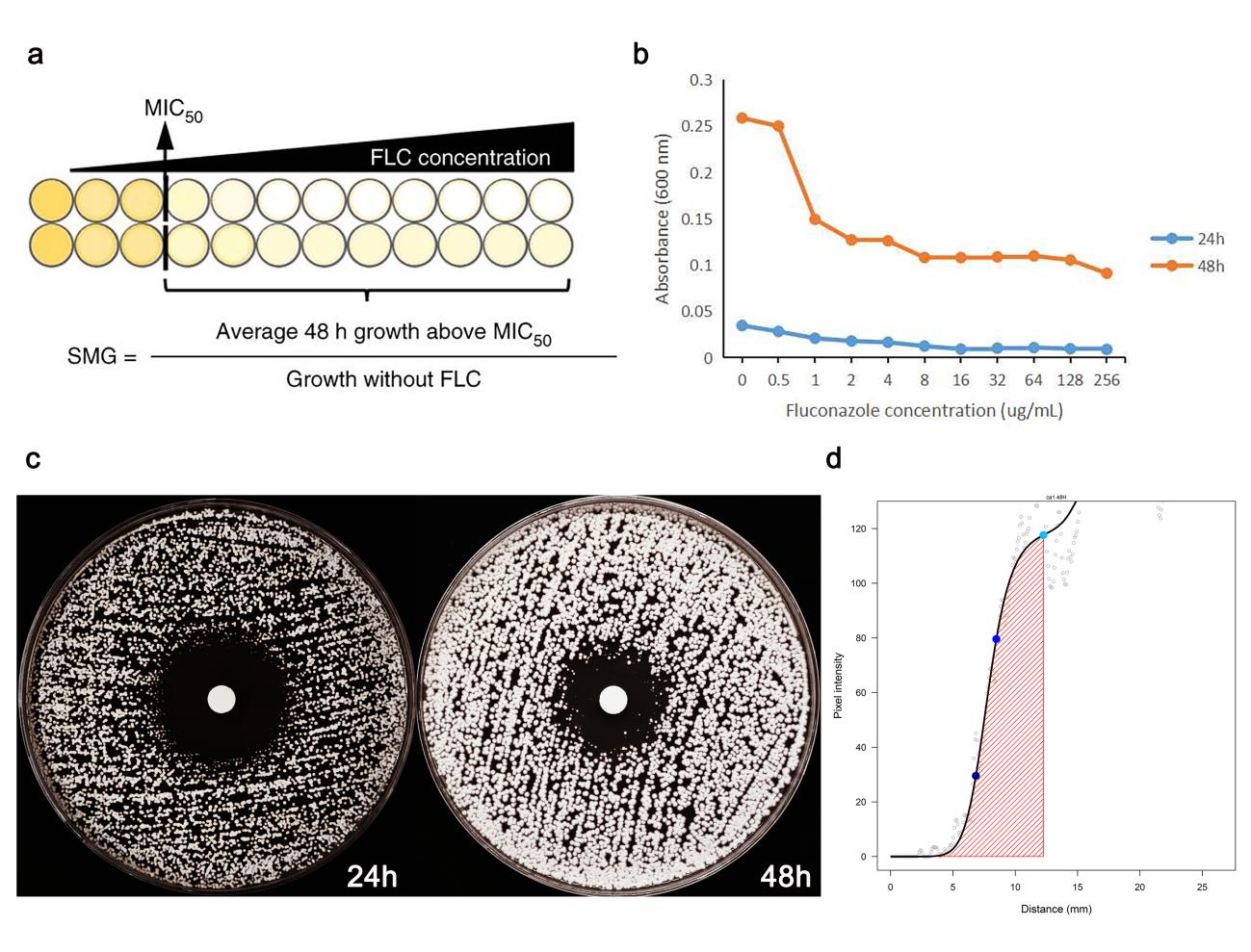


**Supplemenary Fig. 1 |** Measuring drug responses in liquid broth microdilution assays and in disk diffusion assays. (**a)** Illustration of MIC and supra-MIC growth (SMG) calculations^1^. (**b)** Line graph of liquid broth microdilution assays: The MIC50 was determined at 24 hours as the FLC concentration inhibiting 50% growth compared to no drug. SMG was calculated as the average growth above MIC, divided by growth without drug. FLC concentrations ranged from 0 to 256ug/mL in two-fold dilutions. (**c)** Disk diffusion assays of Ca1 at 24h and 48h. (**d)** The result of diskImageR analysis: The average radius (RAD) indicates the mm distance at 20%, 50%, or 80% growth reduction (light, medium, dark blue dots). The FoG is the ratio of the area under the curve (red) at the RAD threshold to the maximum area.

1. Rosenberg A, et al. Antifungal tolerance is a subpopulation effect distinct from resistance and is associated with persistent candidemia. Nature communications 9, 2470 (2018).

**Supplemenary Fig. 2**


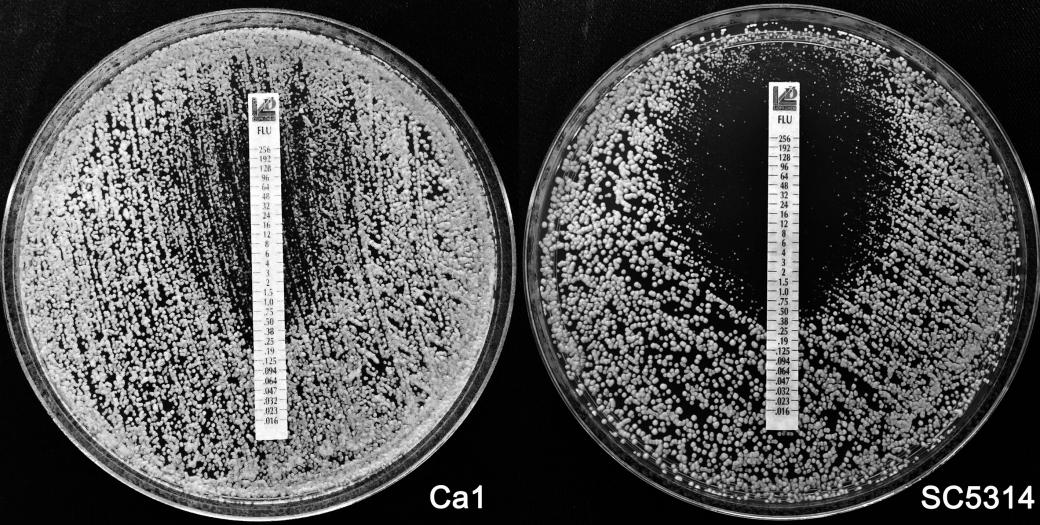


**Supplemenary Fig. 2 |** Comparative E-test results between Ca1 and SC5314. The colonies within the inhibition zone of Ca1 are significantly more than those of SC5314. This phenotypic discrepancy is consistent with their SMG and FoG values.

**Supplemenary Fig. 3**


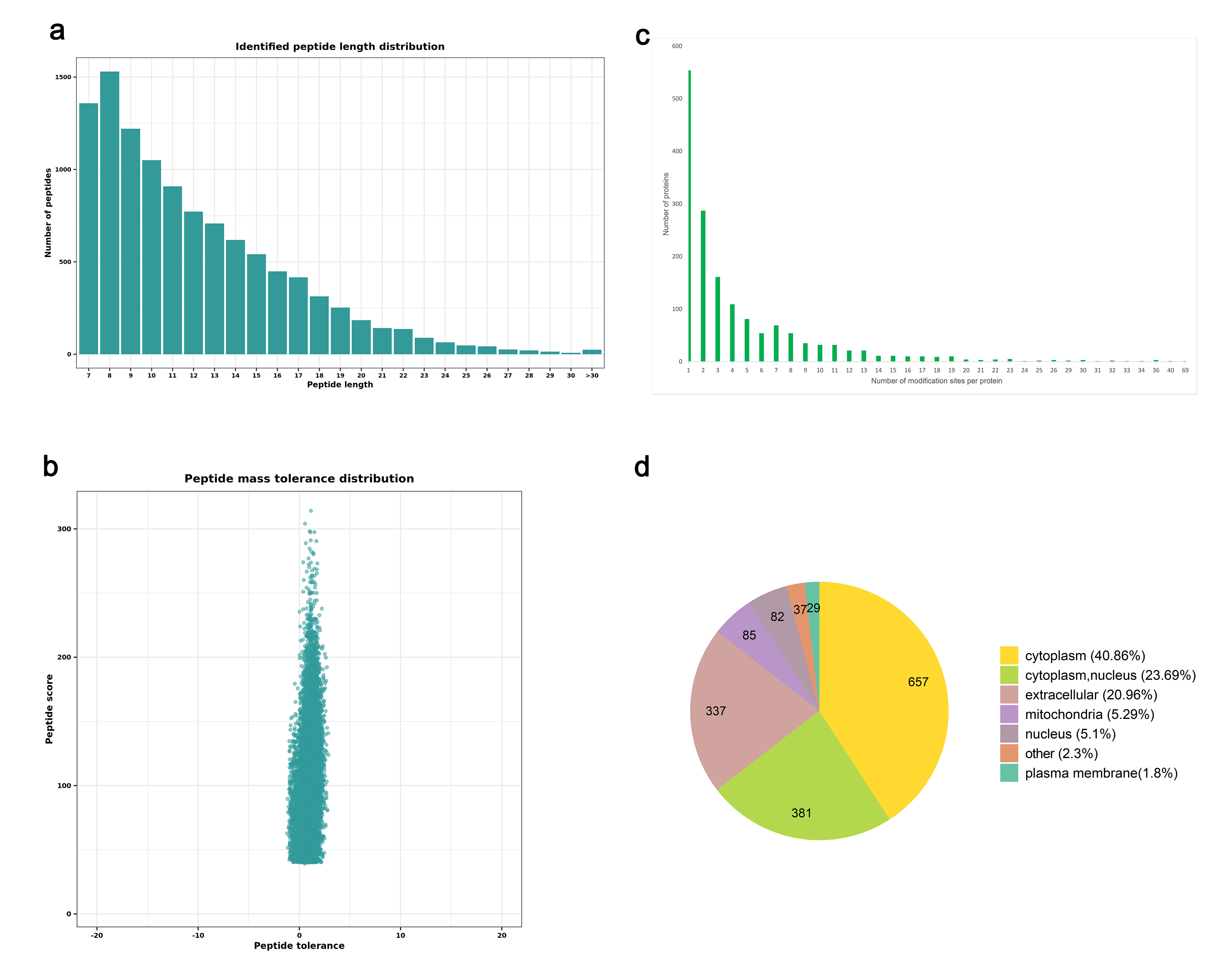


**Supplemenary Fig. 3** | QC validation of MS data and subcellular localization analysis. (**a**) Peptide length distribution. (**b**) Mass error distribution of all identified peptides. (**c**) Number of modification sites per protein. (**d**) Subcellular localization of Kla proteins.


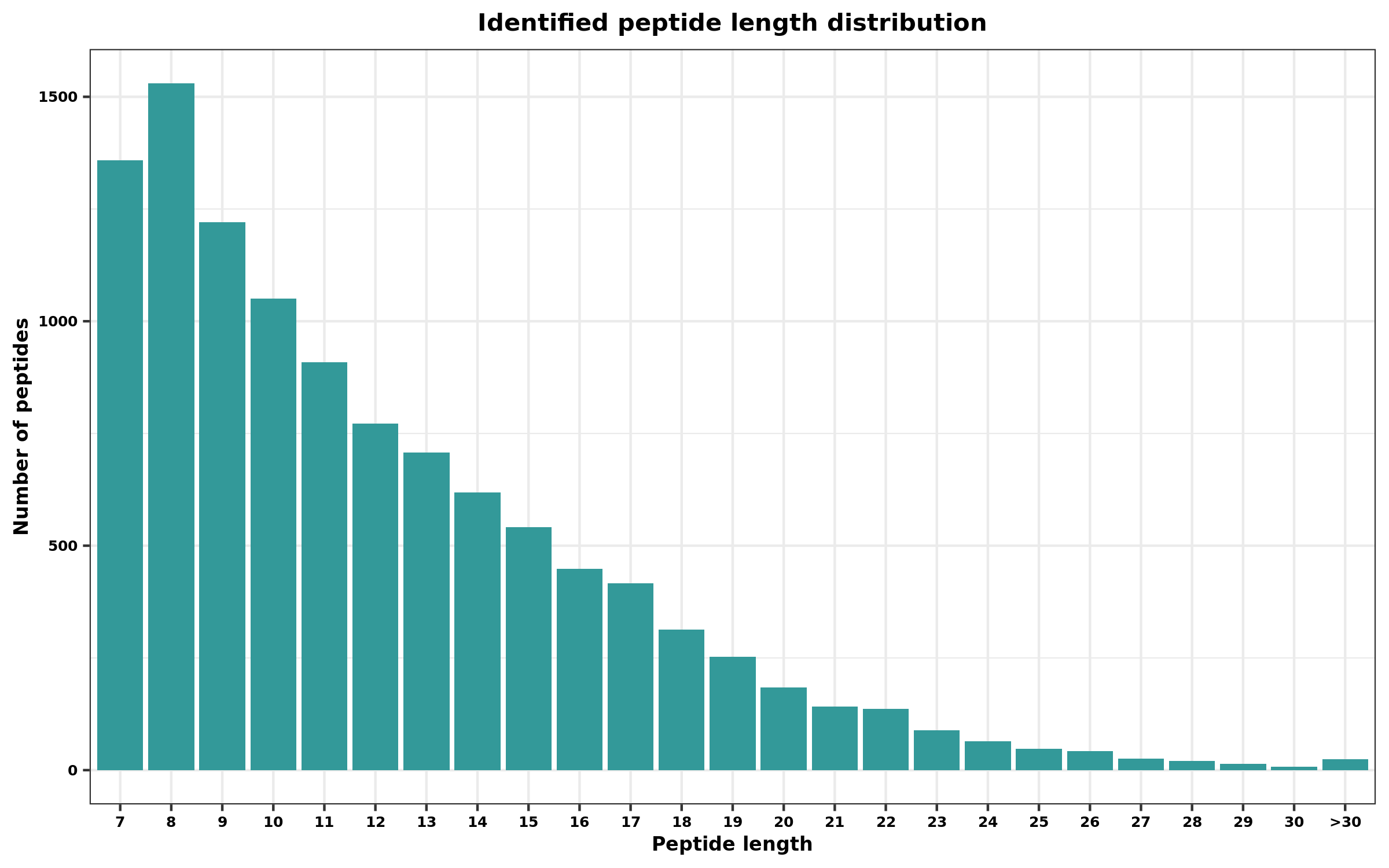


**Supplemenary Fig. 3** (**a**) Peptide length distribution.


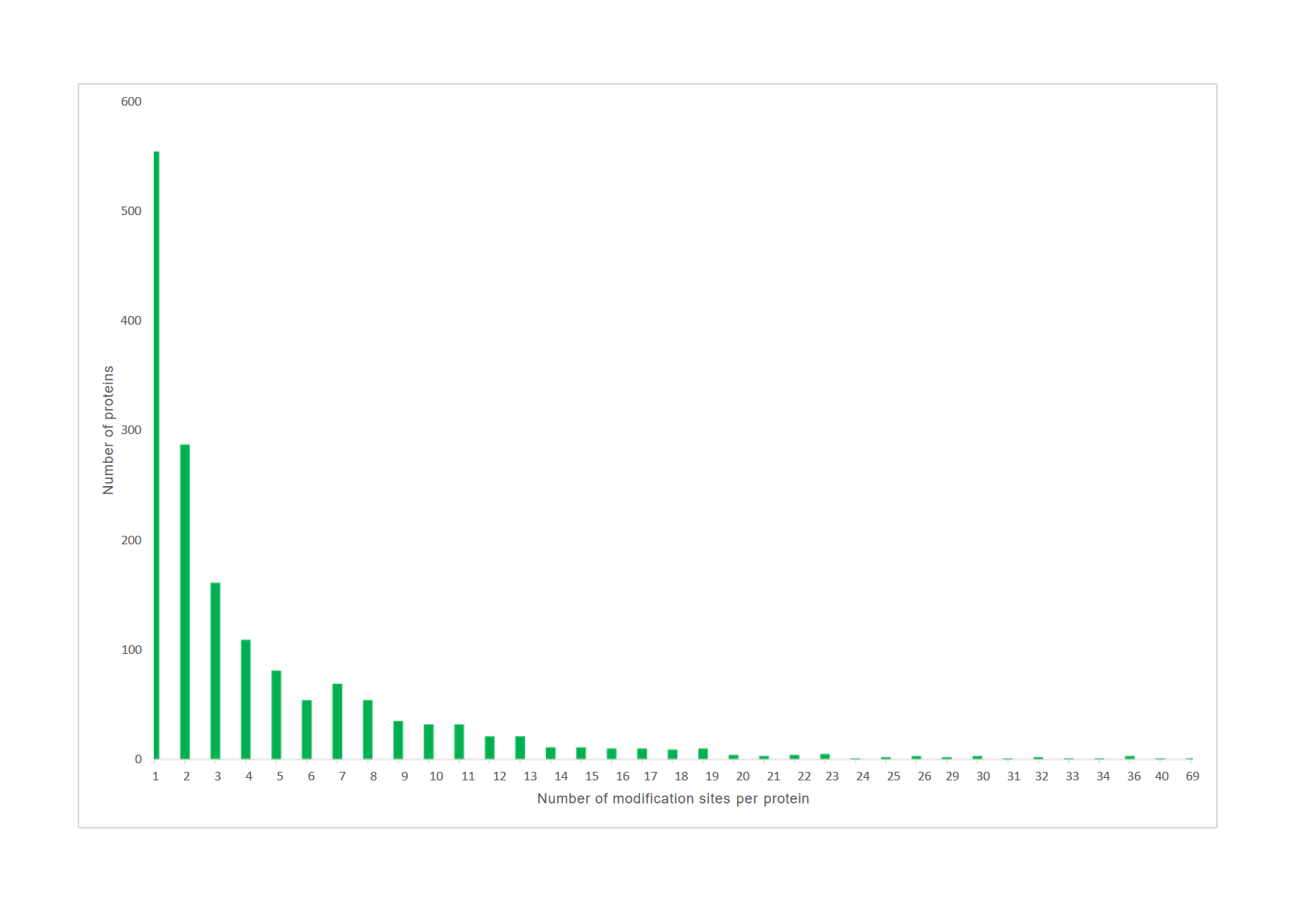


**Supplemenary Fig. 3** (**b**) Mass error distribution of all identified peptides.


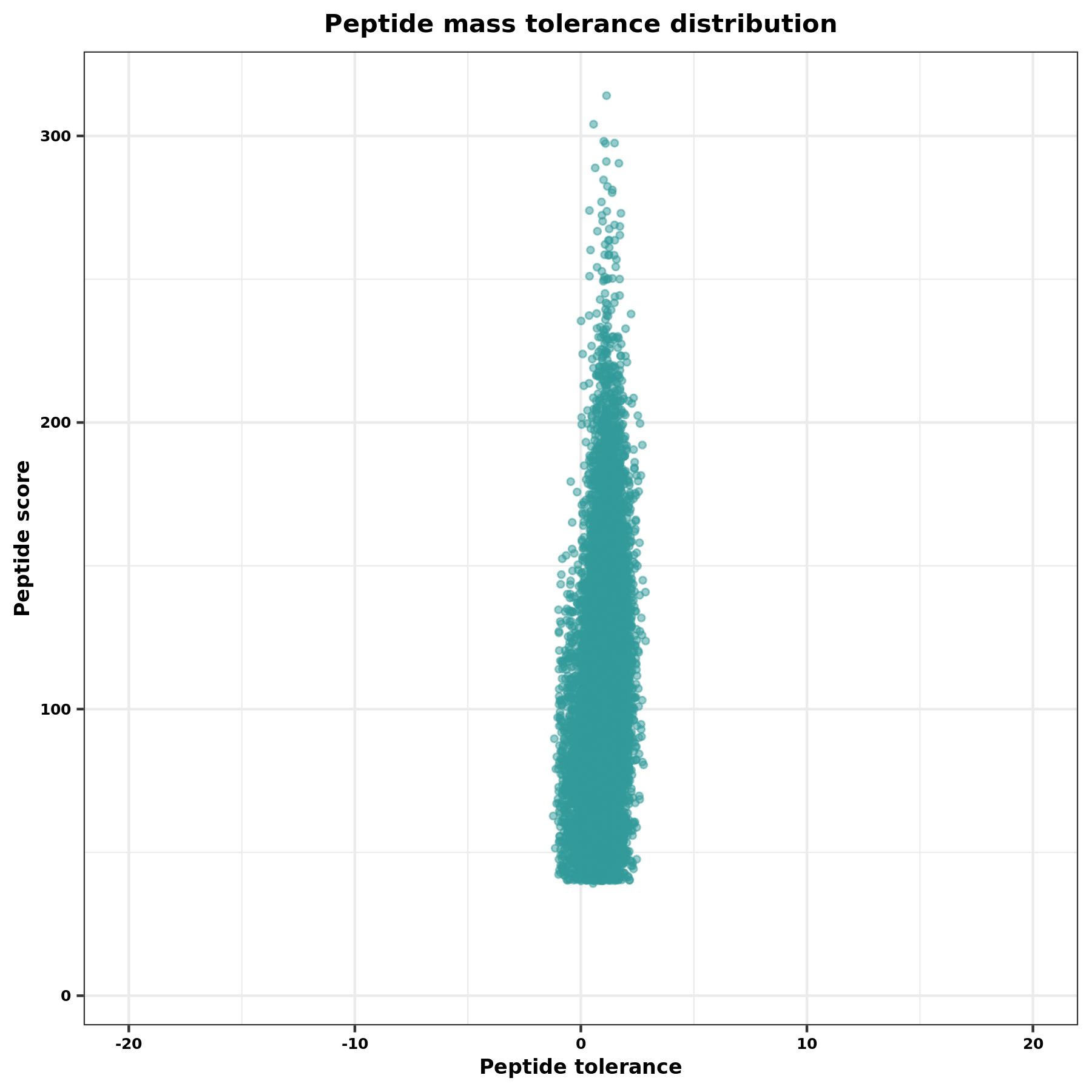


**Supplemenary Fig. 3** (**c**) Number of modification sites per protein.


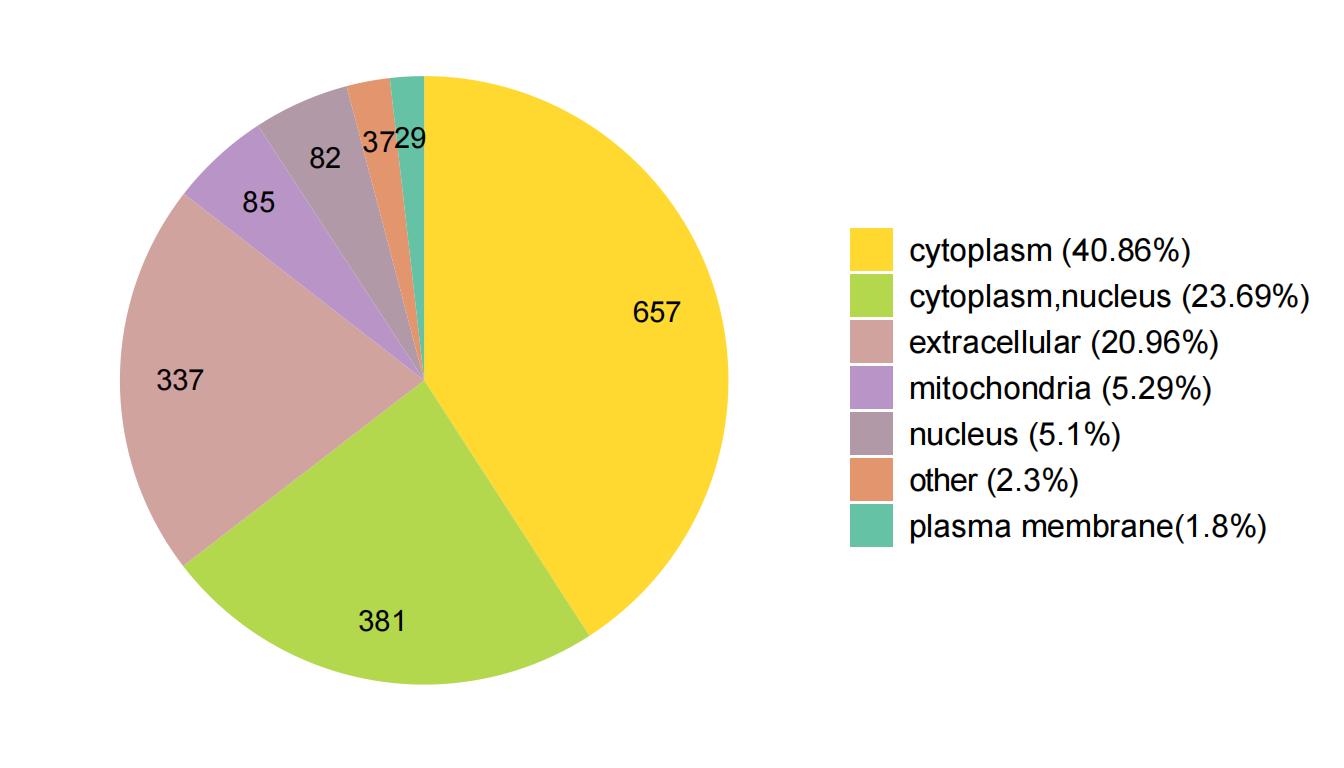


**Supplemenary Fig. 3** (**d**) Subcellular localization of Kla proteins.

**Supplemenary Fig. 4**


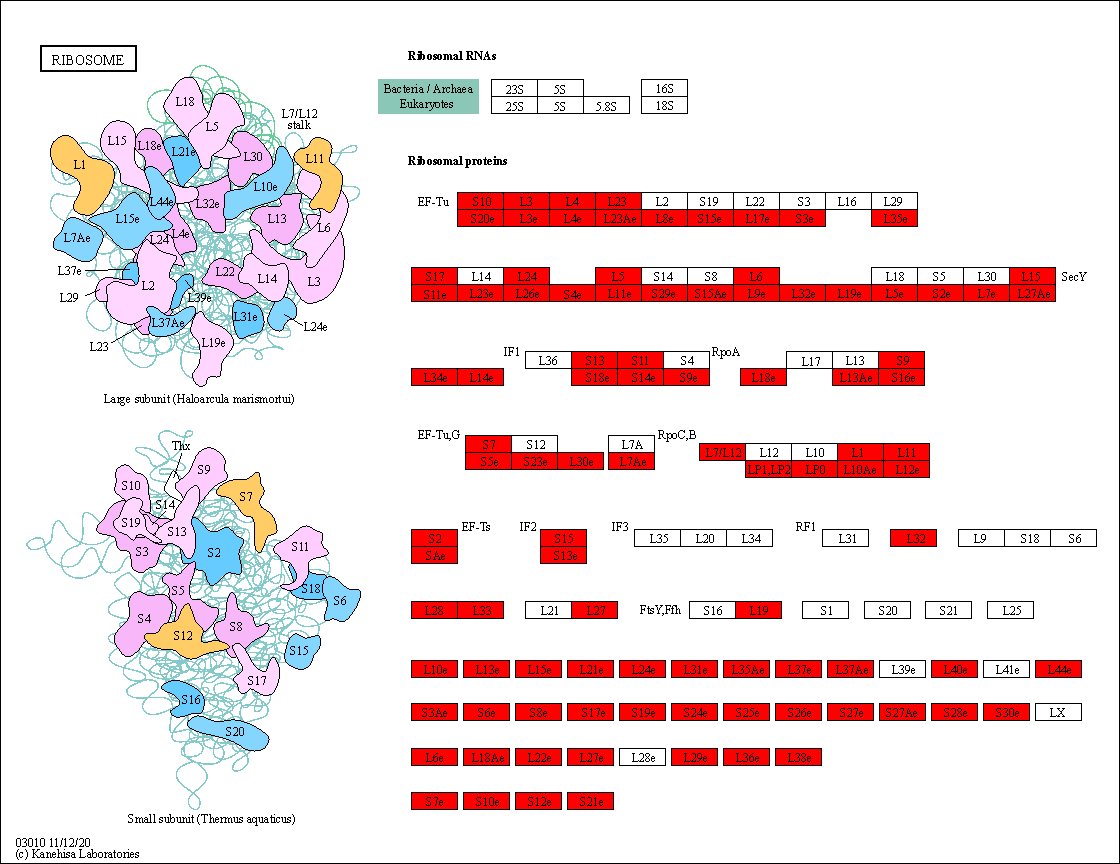


**Supplemenary Fig. 4 |** KEGG pathway enrichment analysis of the identified modified proteins in ribosome. The proteins in red were identified in ribosome of this study.

**Supplemenary Fig. 5**


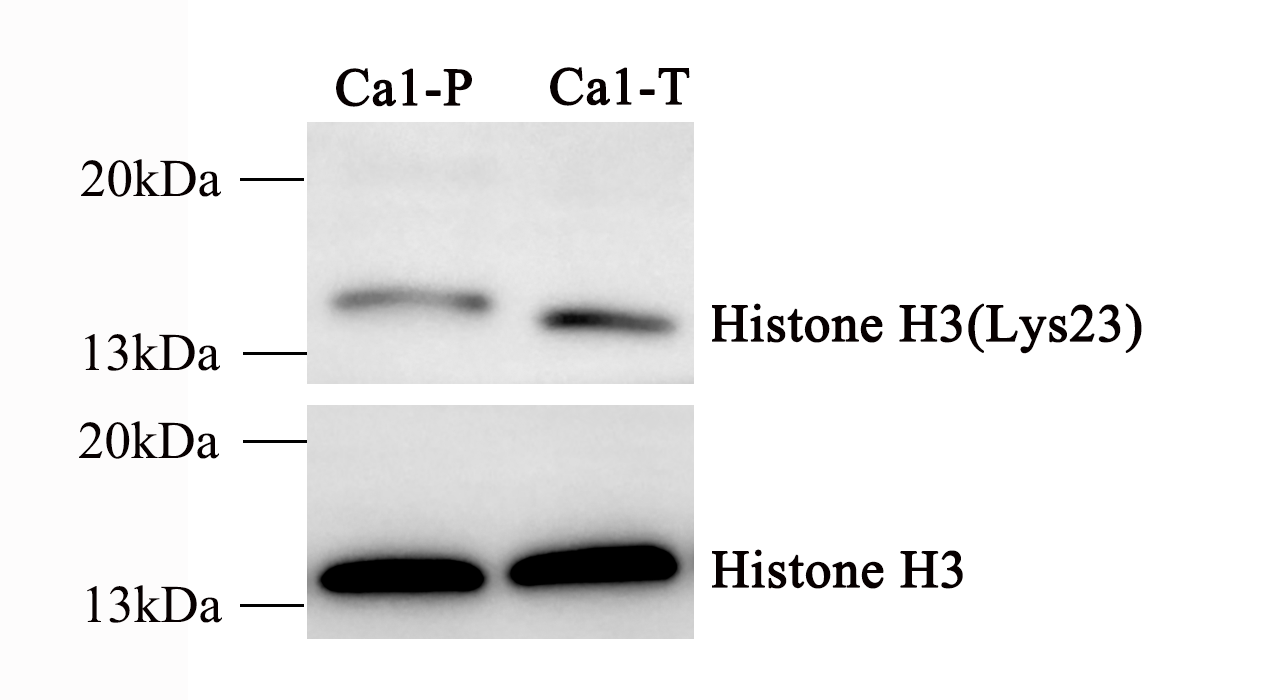


**Supplemenary Fig. 5 |** Immunoblot analysis of Kla at the H3K23 site. The results demonstrate a significant elevation in lactylation levels at the H3K23 site in tolerant cells compared to parental cells (*p*<0.01), with no observable signal saturation across biological replicates (n=3).

**Supplemenary Fig. 6**


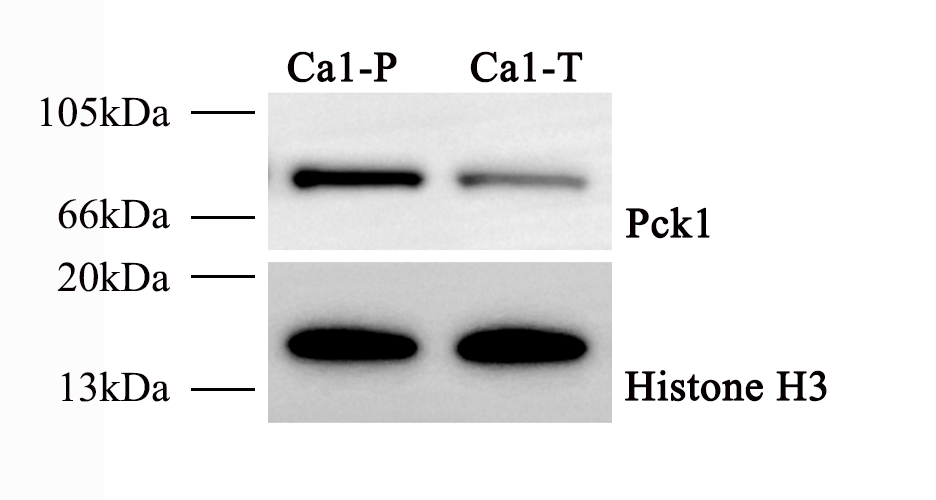


**Supplemenary Fig. 6 |** Western blot analysis of Pck1 expression in fluconazole-tolerant cells (Ca1-T) and parental cells (Ca1-P). The results show a notable decrease in Pck1 expression in tolerant cells (Ca1-T) compared to parental cells (Ca1-P). Histone H3 was used as a loading control.
